# Making science count in government

**DOI:** 10.7554/eLife.01061

**Published:** 2013-07-02

**Authors:** Ian Boyd

**Affiliations:** Department of Environment, Food and Rural Affairs, London, United Kingdomian.boyd@defra.gsi.gov.uk

**Keywords:** Point of view, science policy, bovine TB, DEFRA, funding

## Abstract

Science is an essential component of policy-making in most areas of government, but the scientific community does not always understand its role in this process.

Scientists do not make government policy, and quite rightly they are not expected to, so should they be surprised when policy deviates from what they understand to be the evidence? Many scientists (including social scientists) make an essential contribution to decision-making within government but recent experience suggests to me that there can be confusion amongst some scientists about their role and also about how they can bring their influence to bear most effectively.

Before becoming a Chief Scientific Adviser in UK government I was a full-time professor at the University of St Andrews, and I probably had a fairly typical scientist's view of policy. Experience within my discipline allowed me to speak with some authority on whether policy was generally consistent with current scientific knowledge, and whether the information being used was robust. I also had some specialist knowledge that allowed me to provide direct advice about some specific policy decisions. I often held a view of whether policy was taking a right or wrong direction when the subject was within my own self-appointed area of knowledge but I chose to be agnostic when it was not.

As I now realise there are two difficulties with this position that require careful management. The first is the issue of where authoritative comment stops and political point of view starts; and the second is that any position taken by a scientist is usually a low-dimensional view of a multi-dimensional (or complex) problem. Together, these have a tendency to create a gap between the aspirational views of what policy outcomes should look like, often promoted by the idealised views of scientists, and what these outcomes really look like once they have been through the mangle of policy development.

This can be the source of the dissatisfaction some scientists have with government. Sometimes, often mistakenly, scientists think they are not being listened to and they can quickly change from being a dispassionate commentator to being an agitator when it appears that insufficient weight has been given to scientific evidence. This sets up an unsatisfactory dynamic between scientists and policy-makers that can also involve politicians. It also promotes the suspicion there is among policy-makers that scientists are not as independent as they claim. For example, it is very easy to be drawn in to promoting one's own solutions because of the funding implications.

**Figure 1.**
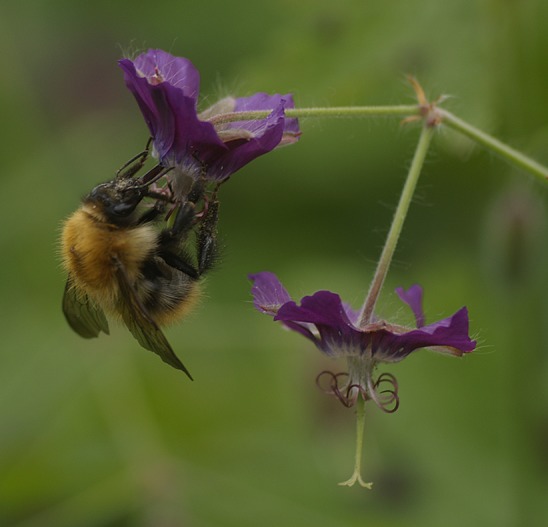
Recent debates about the impact of neonicotinoid pesticides on bees and other pollinators illustrate the complexity of the interface between science and government policy. This photograph shows a common carder bee (*Bombus pascuorum*) feeding on a dusky cranesbill (*Geranium phaeum*).

PHOTOGRAPH: IAN BOYD

Strictly speaking, the role of science should be to provide information to those having to make decisions, including the public, and to ensure that the uncertainties around that information are made clear. When scientists start to stray into providing views about whether decisions based upon the evidence are right or wrong they risk being politicised. In general, it is important for scientists to stick to the evidence and its interpretation.

Strictly speaking, the role of science is to provide information to those having to make decisions.

I will illustrate this through the lens of a recent example involving the control of bovine tuberculosis (TB) in the UK. Controversy has arisen because of the decision to use the culling of badgers as one method to control bovine TB, at least in England. The epidemiology of bovine TB is fiendishly complex and the UK government is struggling to control the disease. It is right for scientists to comment on the information available but several policy options are feasible based upon the evidence. Indeed, if one compares globally among all jurisdictions where bovine TB is a problem, one can find many policy options being pursued. In the UK alone there are different policies being pursued in England (proactive badger culling), Wales (badger vaccination) and Northern Ireland (test and vaccinate), and the Republic of Ireland has opted for yet another option (reactive badger culling). It would be inaccurate to suggest that, based upon the evidence, any of the policy options being pursued across the UK and Ireland is more or less correct; all are possible even if the evidence suggests that some might be more successful than others.

The message in this case is that, based upon the same evidence, it is possible to pursue quite different policy options. Unfortunately, some scientists have been drawn in to the public debate about which policy option is correct. If scientists start to say one or other option is right or wrong then they are beginning to take the position of politicians and they devalue the scientific evidence they claim to present.

Some important mechanisms exist to help scientists convey their views to government. Science advisory committees are an essential mechanism to achieve this and most of these recruit their membership through open competition with independent oversight and scrutiny. Although in some cases membership of these committees means scientists agree to abide by the rules of consensus and collective opinion, the way in which committee advice is published or delivered to politicians means that dissenting, or minority, views can still be expressed. Many scientists show a very high level of commitment to such advisory activities, and their specialist knowledge can have considerable impact on decisions and policies, but the academic community needs to provide greater recognition of this contribution.

If scientists make value judgements outside this framework they risk confusing the public, policy-makers and politicians about the difference between scientific opinion and personal belief. The grey area in which there is scientific uncertainty is somewhere that scientists need to tread very carefully. On the one hand, those making the decisions—the elected politicians—will benefit from well-formed opinion to guide them through this tricky region at the science-policy interface, but scientists should not be surprised if politicians place considerably more weight upon other factors when key decisions have to be made without the support of a strong evidence base.

This highlights a further issue of confusion often prevalent among scientists and that politicians often perpetuate when challenged—the belief that all decisions need to be evidence-based. My experience is that most policy decisions are informed to greater or lesser degrees by evidence but rarely is it practical or even desirable to base decisions entirely upon scientific evidence. The world does not stop at the point where scientific certainty ends, and those implementing policy usually have no choice but to continue making decisions and implementing actions when there is scientific uncertainty. This perhaps represents the greatest disparity between the aspirations of scientists and the reality faced by policy-makers. Moreover, the clarion call from scientists ‘we need to do more research’ is guaranteed to boil the blood of some policy-makers, especially when past investment in science has, if anything, added to the level of apparent scientific uncertainty. The integrity of science starts to be undermined when scientists themselves suggest that the grey area of uncertainty should be occupied by them alone rather than being shared with those implementing policy. This has happened in many of the most controversial areas in my portfolio including bovine TB, pesticides and marine management.

One of the most insidious trends in these and other areas is towards extreme precaution, often built around an extreme interpretation of the precautionary principle. Some scientists can exacerbate this trend towards hazard-based policy-making by failing to provide balanced assessments of the true risks associated with different options, so that the public and policy-makers can themselves make informed decisions.

As a Chief Scientific Adviser, one of my principal roles is to ensure that there is a constructive dialogue between the scientific community and the policy-makers (and those who implement policy) within this grey area of uncertainty. Part of this role is also to make clear where the certainty of the scientific evidence stops and where uncertainty begins. This interfacing role is challenged by two main features of the process.

First, there can be disagreement about what constitutes certainty in the evidence and this is especially true in the environmental sciences where most of my responsibilities sit. Many scientists are excessively optimistic about the strength of evidence supporting particular points of view. In common with preclinical studies (Begley, 2013), the literature on the environmental impact of pesticides has many features that suggest decades of systematic bias in how studies have been commissioned, constructed and published mean that it is nearly impossible to form a clear scientific opinion. This has been illustrated most recently by issues around the effects of neonicotinoid pesticides on pollinators and especially bees (Dicks, 2013).

Second, once a direction of travel for policy has been agreed, if evidence emerges showing that the direction of travel is wrong it can be very difficult to change the policy. There is path-dependency in policy that is very difficult to counteract even where scientific evidence is very strong.

Both these factors play off one another to create conflict between scientists and policy-makers and, in the worst cases, can lead to a breakdown of the relationship and chronically deep-seated mistrust of scientists that can undermine the delicate foundation upon which science builds relevance within the policy-making environment. There is no imperative for science to be included within policy-making and, because of this, flagship issues where the relationship begins to break down, as happened to some extent with the management of bovine TB in England, could set back the cause of science in government across a much wider horizon than just the immediate issue. Scientists involved in any particular issue need to understand the ramifications of their views for quite unrelated areas of policy.

In spite of this, my experience is that government strives to build policies around scientific evidence to an extent that is often under-appreciated by scientists and the public. I have been impressed by the extent to which politicians and policy officials listen carefully to what science says on difficult subjects and often use scientific evidence as the hub around which all decisions are made. But I have also been surprised by just how constrained politicians and policy officials are in the flexibility they have for making decisions. Policy-making is a messy, sometimes chaotic, process because it needs to include social, electoral, ethical, cultural, practical, legal and economic considerations in addition to scientific evidence.

I have been impressed by the extent to which politicians and policy officials listen carefully to what science says on difficult subjects.

The scientific community needs to build a strong sense about how it fits in to this complex mixture to ensure that its contribution to future decisions can be maximised. This means sticking to the evidence and describing clearly what it does and does not say; expressing the balance of risk associated with one or other policy option and avoiding suggesting that policies are either right or wrong; and being willing to make the voice of science heard by engaging with the mechanisms already available through science advisory committees, by working with embedded advisers (such as myself), and by being the voice of reason, rather than dissent, in the public arena.
